# Chromothripsis is correlated with reduced cytotoxic immune infiltration and diminished responsiveness to checkpoint blockade immunotherapy

**DOI:** 10.7150/thno.81350

**Published:** 2023-02-27

**Authors:** Han Chu, Zheng Jin, Jia-nan Cheng, Qingzhu Jia, Bo Zhu, Haoyang Cai

**Affiliations:** 1 Center of Growth, Metabolism, and Aging, Key Laboratory of Bio-Resources and Eco-Environment, College of Life Sciences, Sichuan University, Chengdu, China; 2 Department of Oncology, Xinqiao Hospital, Army Medical University, Chongqing, China; 3 Research Institute, GloriousMed Clinical Laboratory (Shanghai) Co., Ltd, Shanghai, China; 4 Chongqing Key Laboratory of Immunotherapy, Chongqing, China

**Keywords:** Chromothripsis, checkpoint blockade immunotherapy, biomarkers, cytotoxic immune infiltration, responsiveness

## Abstract

**Background:** Chromothripsis caused massive, clustered genomic rearrangements is prevalent in cancer and is considered a new paradigm for tumorigenesis and progression. In this study, we investigated the association among chromothripsis, anti-tumor immune responses, and responsiveness to immune checkpoint blockade (ICB).

**Methods:** Quantification of immune cell infiltration and functional enrichment of immune-related signaling pathways were performed in the discovery set (n = 9403) and the validation set (n = 1140). we investigated the association between chromothripsis and anti-tumor immune responses. In the immunotherapy cohort, copy number alteration-based chromothripsis scores (CPSs) were introduced to assess the extent of chromothripsis to evaluate its association with responsiveness to ICB.

**Results:** In the discovery set and the validation set, the ratios of CD8^+^ T cells to Tregs, TAMs, and MDSCs were significantly lower in tumors with chromothripsis (*P* = 1.5 × 10^-13^, *P* = 5.4 × 10^-8^, and *P* = 1.2 × 10^-4^, respectively, TCGA; *P* = 1.0 × 10^-13^, *P* = 3.6 × 10^-15^, and *P* = 3.3 × 10^-3^, respectively, PCAWG). The relevant pathways underlying the antitumor immune effect were significantly enriched in tumors without chromothripsis. Chromothripsis can be used as an independent predictor, and patients with low-CPSs experienced longer overall survival (OS) after immunotherapy [HR, 1.90; 95% confidence interval, 1.10-3.28; *P* = 0.019].

**Conclusions:** Our findings highlight the reduced cytotoxic immune infiltration in tumors with chromothripsis and enhanced immunosuppression in the tumor microenvironment. Chromothripsis can thus be used as a potential indicator to help identify patients who will respond to ICB, which could complement established biomarkers.

## Introduction

Chromothripsis is typically associated with massive genomic rearrangements accompanied by copy number alterations in a small region of one or several chromosomes 1, 2. This catastrophic event is caused by broken chromosome segments being randomly stitched together by the DNA repair machinery to facilitate cell survival after a huge disruption to the cell genome (massive breakage of chromosomes) 3. However, the causative force of this physical chromosomal damage is unclear 4-6. The definition of chromothripsis and an accurate description of its characteristics are essential for the identification of chromothripsis events. Rigorous judgment criteria have been proposed and explained by Korbel and Campbell 2. A) Breakpoints on chromosomes are clustered. B) The copy number oscillation changes are regular. C) Heterozygous deletion regions and heterozygous regions are spaced apart from each other. D) Those affected by chromothripsis are usually chromatids. E) The joining of DNA fragments is random, i.e., there is no directional preference for joining of fragments. F) The order in which broken DNA fragments are rejoined together is also random, i.e., the distance between the two breakpoints involved in each rearrangement is random. ShatterSeek 7 (whole-genome sequencing-based data) and CTLPScanner 8 (microarray-based data) are currently available for chromothripsis detection and analysis. The classical hypothesis of tumorigenesis and progression assumes that tumorigenesis is a progressive process; as such, tumor precursor cells require cumulative mutations in multiple key genes to acquire a growth advantage, but the concept of chromothripsis challenges this. Chromothripsis might allow for the simultaneous occurrence of oncogenic fusion/amplification and the loss of tumor suppressor genes, which could accelerate the tumorigenic process 7, 9, 10. In addition, chromosomal rearrangements in tumors with chromothripsis present opportunities for tumors to evolve faster to adapt to altered growth conditions (e.g., drug resistance acquisition) 10, 11. Chromothripsis is prevalent in tumors, with liposarcoma and osteosarcoma having the highest susceptibility 7, 12. It has also been reported to be associated with poor prognosis in patients with a variety of cancers 13-16.

Chromosomal instability (CIN), a hallmark of chromothripsis, has been extensively studied in terms of its association with immunity 17, 18. Copy number alteration burden, particularly a copy number loss burden, is associated with reduced gene expression with respect to immune-related pathways. The copy number loss burden is also higher in patients who do not respond to immune checkpoint inhibitor therapy 19. Chromosome somatic copy number alteration (SCNA) levels are associated with immune escape, and a high SCNA level is associated with poorer patient survival. Further, it has been suggested that a large fraction of canonical chromothripsis events in polyploid tumors are late events 7, 20. This suggests a potential association between chromothripsis and immunity. Chromothripsis is a primary mechanism that accelerates genomic DNA rearrangements and amplification to form circular extrachromosomal DNA (ecDNA) 10. EcDNA is encapsulated in micronuclei, which represent an important source of immunostimulatory DNA 4, 21, 22. This suggests the involvement of chromothripsis in the cGAS-sting pathway, which is a component of innate immunity, through micronucleus formation. In addition, clinical data indicate a higher incidence of chromothripsis in patients exhibiting weaker anti-tumor immune effects 23. Collectively, the association between chromothripsis and antitumor immune response is ambiguous.

Here, our objective was to investigate the relationship among chromothripsis, anti-tumor immune responses, and responsiveness to immune checkpoint blockade (ICB) immunotherapy. In both a discovery and validation dataset, we identified consistently reduced immune cell infiltration in tumors with chromothripsis, along with impaired cytolytic activity. We also explored the association between tumor chromothripsis and broad manifestations in the immune microenvironment. In addition, we constructed chromothripsis prediction models from copy number alteration (CNA) signatures and obtained chromothripsis scores (CPSs) from them to elucidate the relationship between chromothripsis and therapeutic outcomes in patients receiving ICB immunotherapies.

## Results

### Chromothripsis is correlated with reduced cytotoxic immune infiltration in the discovery The Cancer Genome Atlas (TCGA) dataset

To investigate the effect of chromothripsis on the tumor microenvironment, we examined multi-omics data from 24 cancer types (solid tumors only) from TCGA. For copy number profiles derived from the SNP6 microarray, we used CTLPScanner to detect and annotate chromothripsis in patients 8. At the same time, we quantified immune-related features based on gene expression profiles using established methods (see Materials and Methods for details).

**Figure 1:**
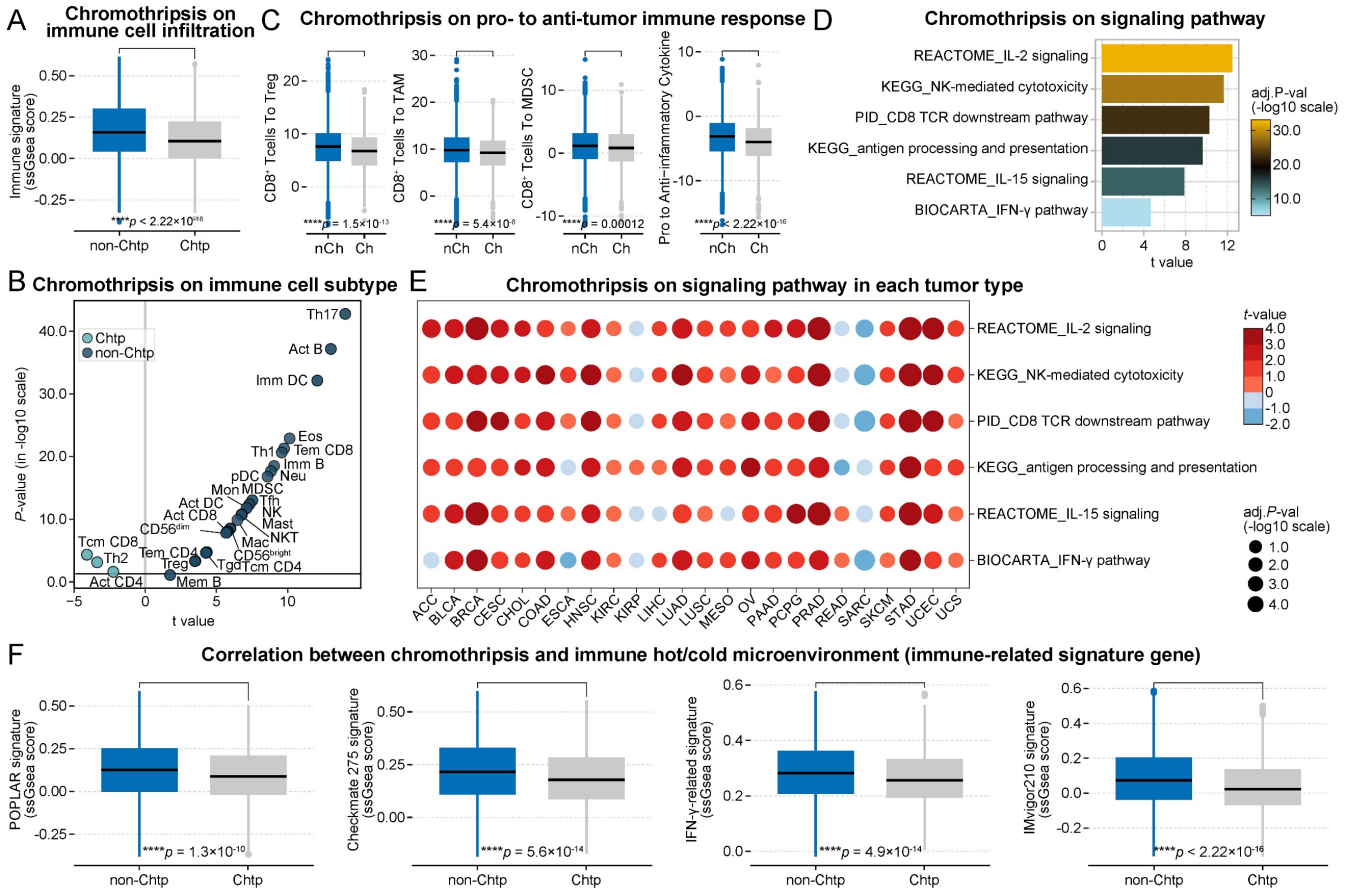
** Association between chromothripsis and cytotoxic immune infiltration in TCGA dataset. (A)** Total immune cell infiltration score (ssgsea score) in chromothripsis (Chtp) and non-chromothripsis (non-Chtp) groups (two-sided Wilcoxon rank sum test). **(B)** Volcano plot showing the enrichment of tumors with chromothripsis (Chtp) and without chromothripsis (non-Chtp) calculated based on the t-value from the Gene Set Variation Analysis (GSVA). **(C)** The ratio of CD8^+^ T cells to Tregs, CD8^+^ T cells to tumor-associated macrophages (TAMs), CD8^+^ T cells to myeloid-derived suppressor cells (MDSCs), and pro- to anti-inflammatory cytokines (geometric mean) in chromothripsis (Ch) and non-chromothripsis (nCh) groups (two-sided Wilcoxon rank sum test). **(D)** Bar plot showing the enrichment of tumor without chromothripsis (non-Chtp) calculated based on the t-value from the GSVA. **(E)** Bubble plot showing the enrichment of tumors without chromothripsis (non-Chtp) calculated based on the t-value from the GSVA (24 cancer types). The size of the circles indicates the adjusted log-rank p-value, and the color indicates the t-value. **(F)** Immune checkpoint blockade (ICB)-responsiveness score of the tumor microenvironment in chromothripsis (Chtp) and non-chromothripsis (non-Chtp) groups (two-sided Wilcoxon rank sum test).

Patients were divided into two groups based on the occurrence of chromothripsis, where the total immune cell infiltration score was significantly lower in the chromothripsis group than in the non-chromothripsis group (median 1.58 × 10^-1^ vs 1.05 × 10^-1^, P < 2.2 × 10^-16^, **Figure 1A**). The enrichment of 28 tumor-infiltrating immune cells in the tumor (**Figure 1B**, see Table S1A for details) was used to further characterize the changes in the microenvironmental composition of tumors with chromothripsis 24. First, we observed that cytotoxic lymphocytes (CD8^+^ T cells and natural killer (NK) cells), which are considered the primary executor of antitumor immunity 25, were enriched in tumors without chromothripsis. Moreover, we found that immunosuppression-associated tumor-infiltrating myeloid-derived suppressor cells (MDSCs), tumor-associated macrophages (TAMs), and regulatory T cells (Tregs) 26 were enriched in the tumors without chromothripsis.

In accordance with the decrease in tumor-infiltrating cytotoxic and immunosuppressive cells in tumors with chromothripsis, we then investigated the pro-tumorigenic versus anti-tumorigenic properties of the immune microenvironment, as previously reported 27. The ratios of CD8^+^ T cells to Tregs, TAMs, and MDSCs were significantly lower in tumors with chromothripsis (median 7.60 vs 6.79, *P* = 1.5 × 10^-13^; median 9.77 vs 9.24, *P* = 5.4 × 10^-8^; median 1.16 vs 8.21 × 10^-1^, *P* = 1.2 × 10^-4^, respectively; **Figure 1C**), suggesting an immunosuppressive microenvironment in these tumors. In addition, the expression ratio of proinflammatory cytokines (IFN-γ, IL-1A, IL-1B, and IL-2, markers of immune stimulation) to immunosuppressive molecules (IL-4, IL-10, IL-11, and TGFB1) was significantly reduced in tumors with chromothripsis (median -3.12 vs -4.00, *P* < 2.2 × 10^-16^) 27, also implying that pro-immunogenic responses are relatively reduced in tumors with chromothripsis.

Further, Gene Set Variation Analysis (GSVA) revealed partial signaling pathways involved in anti-tumor immune effects, including antigen presentation (*t* = 9.61, *P* = 6.4 × 10^-21^, antigen processing and presentation), antigen recognition (*t* = 9.49, *P* = 2.1 × 10^-20^, CD8 TCR downstream pathway), effector cell activation (*t* = 12.47, *P* = 7.5 × 10^-34^, IL-2 signaling; *t* = 7.87, *P* = 1.6 × 10^-14^, IL-15 signaling), and immune-mediated cytotoxicity (*t* = 11.64, *P* = 8.1 × 10^-30^, NK-mediated cytotoxicity; *t* = 4.64, *P* = 7.1 × 10^-6^, IFN-γ pathway) were significantly enriched in tumors without chromothripsis (**Figure 1D**, see Table S2A for details). Gene Set Enrichment Analysis (GSEA) yielded consistent results (**Figure S1**). The analysis of individual tumor types also resulted in generally consistent results (**Figure 1E**, see Table S2B for details), with the notable exception of osteosarcoma, which develops from mesenchymal cells. These data indicated that tumors with chromothripsis are more prone to immune escape than those without. Using multiple gene signatures from clinical trials or those widely used to evaluate the ICB-responsiveness of the tumor microenvironment 28-31, we further revealed that the microenvironment of tumors with chromothripsis was relatively less sensitive to ICB treatment (POPLAR signature, median 1.27 × 10^-1^ vs 8.77 × 10^-2^, *P* = 1.3 × 10^-10^; Checkmate 275 signature, median 2.15 × 10^-1^ vs 1.78 × 10^-1^, *P* = 5.6 × 10^-14^; IFN-γ-related signature, median 2.83 × 10^-1^ vs 2.56 × 10^-1^, *P* = 4.9 × 10^-14^; IMvigor210 signature, median 7.30 × 10^-2^ vs 2.28 × 10^-2^, *P* < 2.2 × 10^-16^; **Figure 1F**).

### Chromothripsis is correlated with reduced cytotoxic immune infiltration in the validation Pancancer Analysis of Whole Genomes (PCAWG) dataset

The development of next-generation sequencing technology allows us to easily obtain whole genome sequencing (WGS) data, based on which we can call CNA and Structure Variantion (SV). Using SV information, ShatterSeek can be used to detect and annotate tumor chromothripsis 7. Here, we acquired multi-omics data from the PCAWG project 32 to validate the findings of TCGA dataset.

**Figure 2:**
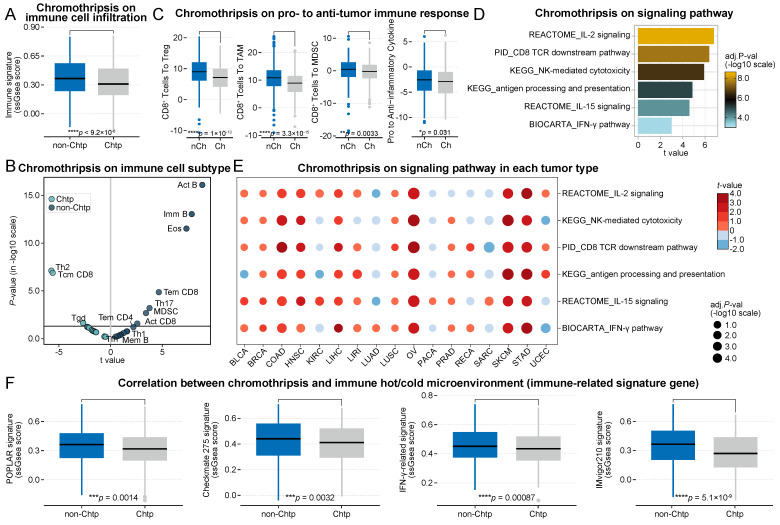
** Association between chromothripsis and cytotoxic immune infiltration in PCAWG dataset. (A)** Total immune cell infiltration scores (ssgsea scores) in chromothripsis (Chtp) and non-chromothripsis (non-Chtp) groups (two-sided Wilcoxon rank sum test). **(B)** Volcano plot for the enrichment tumors with chromothripsis (Chtp) and without chromothripsis (non-Chtp) calculated based on the t-value from the Gene Set Variation Analysis (GSVA). **(C)** Ratio of CD8^+^ T cells to Tregs, CD8^+^ T cells to tumor-associated macrophages (TAMs), CD8^+^ T cells to myeloid-derived suppressor cells (MDSCs), and pro- to anti-inflammatory cytokines (geometric mean) in chromothripsis (Ch) and non-chromothripsis (nCh) groups (two-sided Wilcoxon rank sum test). **(D)** Bar plot showing the enrichment of tumors without chromothripsis (non-Chtp) calculated based on the t-value from the GSVA. **(E)** Bubble plot showing the enrichment of tumors without chromothripsis (non-Chtp) calculated based on the t-value from the GSVA (17 cancer types). The size of the circles indicates the adjusted log-rank p-value, and the color indicates the t-value. **(F)** Immune checkpoint blockade (ICB)-responsiveness score of tumor microenvironment in chromothripsis (Chtp) and non-chromothripsis (non-Chtp) groups (two-sided Wilcoxon rank sum test).

Consistent with the previous results, the total immune cell infiltration score was significantly lower in the chromothripsis group than in the non-chromothripsis group (median 3.66 × 10^-1^ vs 3.09 × 10^-1^, *P* = 9.2 × 10^-6^, **Figure 2A**). In addition, the distribution of tumor-infiltrating immune cell enrichment results was almost unchanged (**Figure 2B**). The ratios of CD8^+^ T cells to Tregs, TAMs, and MDSCs, including the expression ratio of pro-inflammatory cytokines to immunosuppressive molecules, were significantly lower in the chromothripsis group (median 9.00 vs 7.15, *P* = 1.0 × 10^-13^; median 10.87 vs 8.93, *P* = 3.3 × 10^-15^; median 4.70 × 10^-1^ vs -2.00 × 10^-1^, *P* = 3.3 × 10^-3^; median -2.55 vs -2.91 × 10^-1^, *P* = 3.1 × 10^-2^, respectively; **Figure 2C**). The relevant pathways underlying the antitumor immune effect were significantly enriched in tumors without chromothripsis (*t* = 4.87, *P* = 9.4 × 10^-6^, antigen processing and presentation; *t* = 6.37, *P* = 8.4 × 10^-9^, CD8 TCR downstream pathway; *t* = 6.79, *P* = 8.0 × 10^-10^, IL-2 signaling; *t* = 4.60, *P* = 2.9 × 10^-5^, IL-15 signaling; *t* = 5.92, *P* = 4.3 × 10^-9^, NK-mediated cytotoxicity; *t* = 2.99, *P* = 7.2 × 10^-3^, IFN-γ pathway, **Figure 2D**), and the results of GSEA were consistent with this (**Figure S2**). The results of the enrichment analysis for individual cancer types were also largely consistent with the previous data (**Figure 2E**). The tumor microenvironment of the chromothripsis group was significantly less sensitive to ICB treatment than that of the non-chromothripsis group (POPLAR signature, median 3.63 × 10^-1^ vs 3.17 × 10^-1^, *P* = 1.4 × 10^-3^; Checkmate 275 signature, median 4.40 × 10^-1^ vs 4.11 × 10^-1^, *P* = 3.2 × 10^-3^; IFN-γ-related signature, median 4.51 × 10^-1^ vs 4.34 × 10^-1^, *P* = 8.7 × 10^-4^; IMvigor210 signature, median 3.66 × 10^-1^ vs 2.70 × 10^-1^, *P* = 5.1 × 10^-9^; **Figure 2F**), which is consistent with the results obtained from TCGA dataset. The results from the discovery and validation datasets simultaneously show that chromothripsis is associated with a reduction in cytotoxic immune cell infiltration in the tumor microenvironment and that patients with chromothripsis have reduced sensitivity to immunotherapy.

### Association between chromothripsis with genetic features

**Figure 3:**
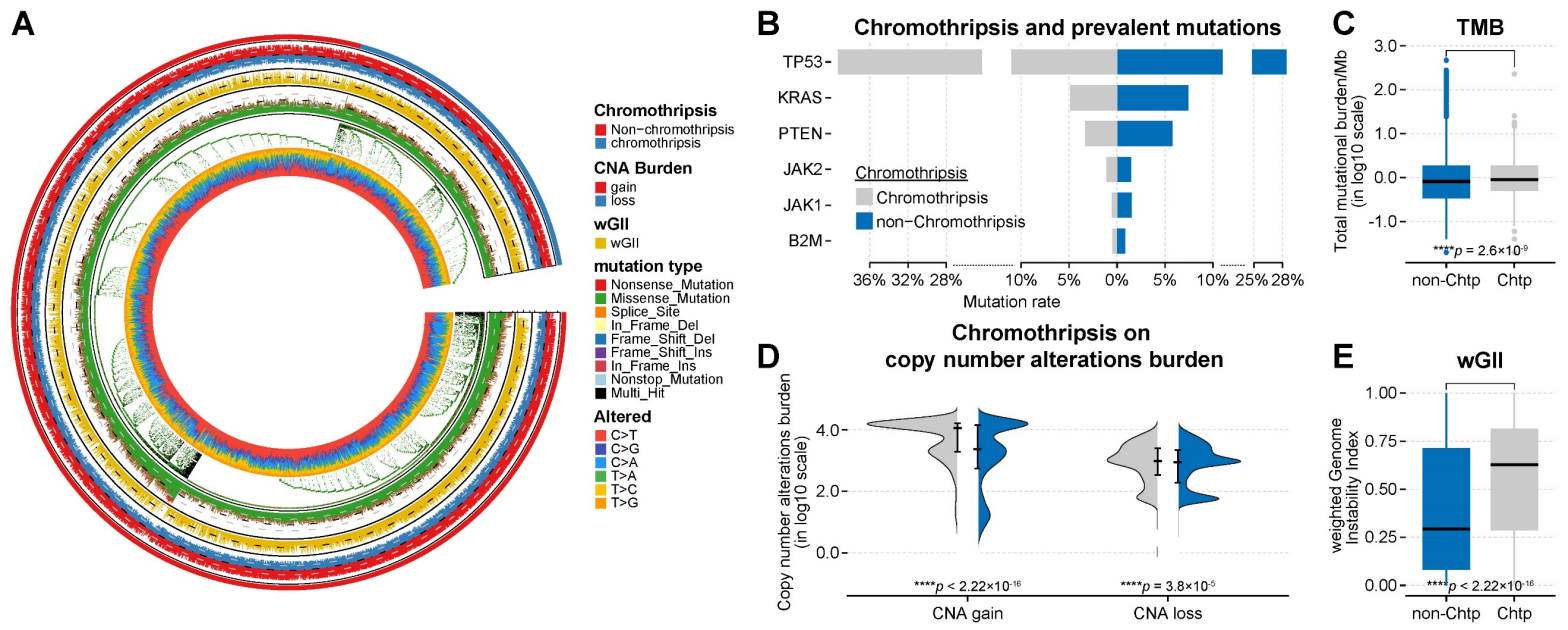
** Association between chromothripsis and other genetic features of cancer in TCGA dataset. (A)** General overview of genetic characteristics of cancer patients in TCGA dataset (including chromothripsis, copy number alteration (CNA) burden, mutation type, and nucleotide alterations). **(B)** Mutation frequency of key genes in chromothripsis and non-chromothripsis groups. **(C)** Total mutational burden/MB in chromothripsis and non-chromothripsis groups (two-sided Wilcoxon rank sum test). **(D)** Violin plot showing copy number alteration burden (including CNA loss and gain) in chromothripsis and non-chromothripsis groups. **(E)** Weighted genome instability index (at te level of somatic copy number alterations (SCNAs)) in chromothripsis and non-chromothripsis groups (two-sided Wilcoxon rank sum test).

In addition to transcriptomic expression profiling, we next focused on genetic features of cancer, particularly immune-related predictors. From patient samples, we obtained the mutation frequencies in ICB-responsiveness-related genes (including *TP53*, *KRAS* 33, *PTEN* 34, *JAK1*/2 35, and *B2M* 36), the tumor mutation burden (TMB) 37, 38, the burden of somatic copy number alterations 19, and the level of somatic copy-number alterations (quantified as weighted genome instability (wGII) 39; see Materials and Methods for details) to comprehensively compare their relationship with chromothripsis. Consistent with previous publications 7, 9, *TP53* mutation frequencies were higher in-patient samples from the chromothripsis group than in those from the non-chromothripsis group in the discovery TCGA dataset. In the pan-cancer scale analysis, the differences in mutation frequencies of the remaining representative genes were much lower than that for *TP53* (see Table S3C for details, **Figure 3B**).

The TMB was modest and significantly higher in tumors with chromothripsis (median -8.62 × 10^-2^ vs 4.58 × 10^-2^, *P* = 2.6 × 10^-9^, **Figure 3C**), suggesting that chromothripsis can lead to an increase in somatic mutations and represents a possible increase in tumor neoantigens. Expectedly, the SCNA levels, represented by wGII, were also significantly higher in tumors with chromothripsis (median 2.94 × 10^-1^ vs 6.28 × 10^-1^, *P* < 2.2 × 10^-16^, **Figure 3E**), which resulted from the massive, clustered genomic rearrangements mediated by chromothripsis. Chromothripsis could thus lead to increased wGII.

**Figure 4:**
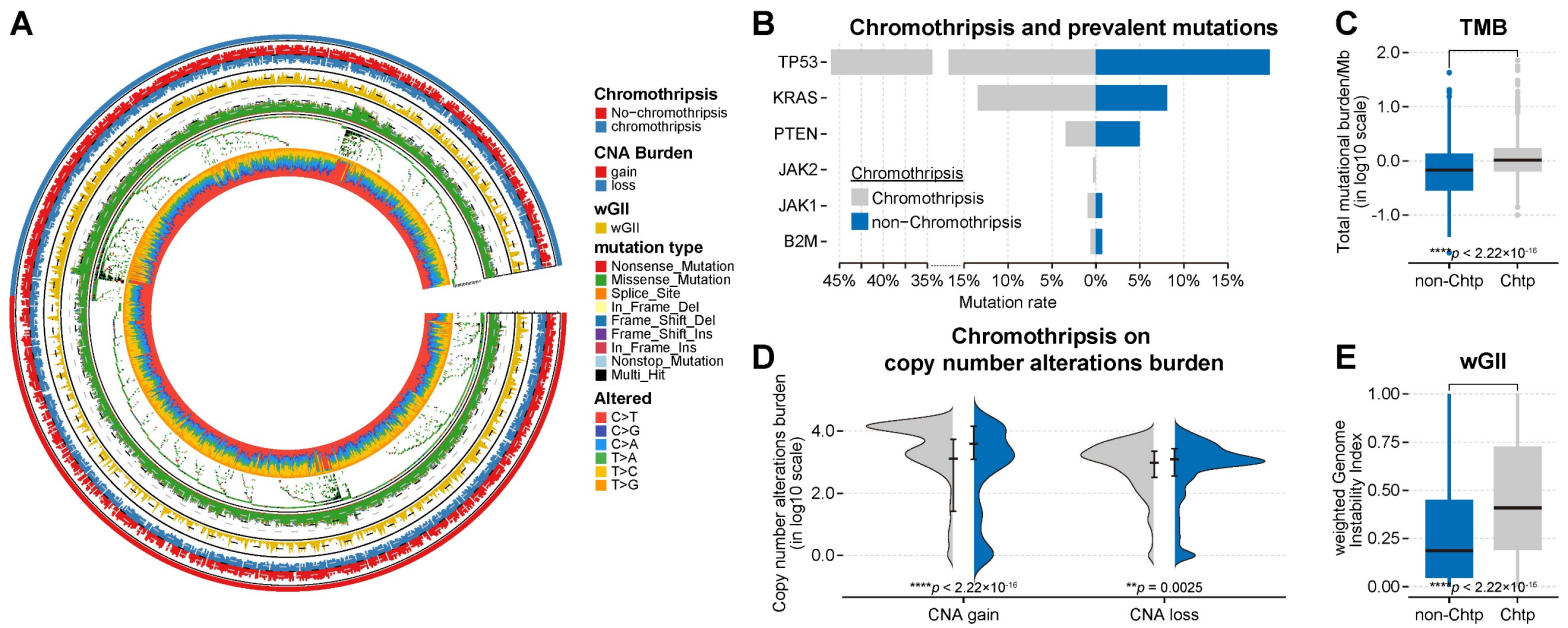
** Association between chromothripsis and other genetic features of cancer in PCAWG dataset. (A)** General overview of genetic characteristics of cancer patients in the PCAWG dataset (including chromothripsis, copy number alteration (CNA) burden, mutation type, and nucleotide alterations). **(B)** Mutation frequency of key genes in chromothripsis and non-chromothripsis groups. **(C)** Total mutational burden/MB in chromothripsis and non-chromothripsis groups (two-sided Wilcoxon rank sum test). **(D)** Violin plot showing copy number alteration burdens (including CNA loss and gain) in chromothripsis and non-chromothripsis groups. **(E)** Weighted genome instability index (at the level of somatic copy number alterations (SCNAs)) in chromothripsis and non-chromothripsis groups (two-sided Wilcoxon rank sum test).

Similarly, we found that the *TP53* mutation frequency in the validation PCAWG dataset was higher in-patient samples with chromothripsis (see Table S3C for details, **Figure 4B**). TP53 malfunction could be a predisposing factor for chromothripsis^4^. Although we found a higher incidence of *TP53* mutations in tumors with chromothripsis in the discovery and validation set, >60% (mean value) of the tumors with chromothripsis never showed *TP53* mutations or amplifications. This suggested that TP53 (and other representative genes) might not be a significant factor in suppressing the anti-tumor immune response in tumors with chromothripsis.

Both TMB and wGII are also significantly increased in the chromothripsis group based on the validation PCAWG dataset (median -1.67 × 10^-1^ vs 1.70 × 10^-2^, *P* < 2.2 × 10^-16^; median 1.86 × 10^-1^ vs 4.09 × 10^-1^, *P* < 2.2 × 10^-16^, **Figure 4C**, **Figure 4E**, respectively). A high TMB was associated with favorable survival outcomes in patients receiving ICB immunotherapy 37, 40, 41. The higher TMB associated with chromothripsis in tumors suggests that TMB might not be a significant factor in suppressing the anti-tumor immune response in tumors with chromothripsis.

In addition, we observed inconsistent results. In the discovery TCGA dataset, the burden of copy number alterations (CNA burden, including CNA loss and CNA gain) was significantly higher in the chromothripsis group (*P* < 2.2 × 10^-16^, *P* = 3.8 × 10^-5^, respectively, **Figure 3D**). In the validation PCAWG dataset, the CNA burden was significantly lower in tumors with chromothripsis (*P* < 2.2 × 10^-16^, *P* = 2.5 × 10^-3^, respectively, **Figure 4D**). There were many possibilities for this difference, and this difference suggested a possible stochastic association between the CNV burden and chromothripsis. The CNV burden cannot be a major factor in the reduced antitumor immune response in tumors with chromothripsis. Collectively, we believed that chromothripsis can be used as an independent predictor.

### Chromothripsis scores predict survival outcomes for patients after immunotherapy

Given the correlation between chromothripsis and ICB-responsiveness features, we next examined whether there was a correlation between chromothripsis and patient survival after immunotherapy. We acquired datasets from three ICB-treated clinical trials, including two melanoma cohorts and one glioma cohort 42-44, for which tumors were available for whole-exome sequencing (WES) data analysis. CN signatures serve as a flexible tool to identify the presence of chromothripsis, with a performance comparable to that of ShatterSeek for both WES and WGS data 13. We further validated the reliability of this tool using both the discovery and validation datasets (TCGA, AUC = 0.81; PCAWG, AUC = 0.89; **Figure S3**). We obtained CPSs based on a CN signature prediction model. Notably, we integrated all three immunotherapy cohorts to expand the sample size and ensure the reliability of results from the prediction model. In addition, we accounted for the TMB, wGII, CNA burden, PD-L1 expression level, and CD8A expression level (see Table S4 for details), which have been described as biomarkers of survival outcomes for patients treated with immunotherapy.

**Figure 5:**
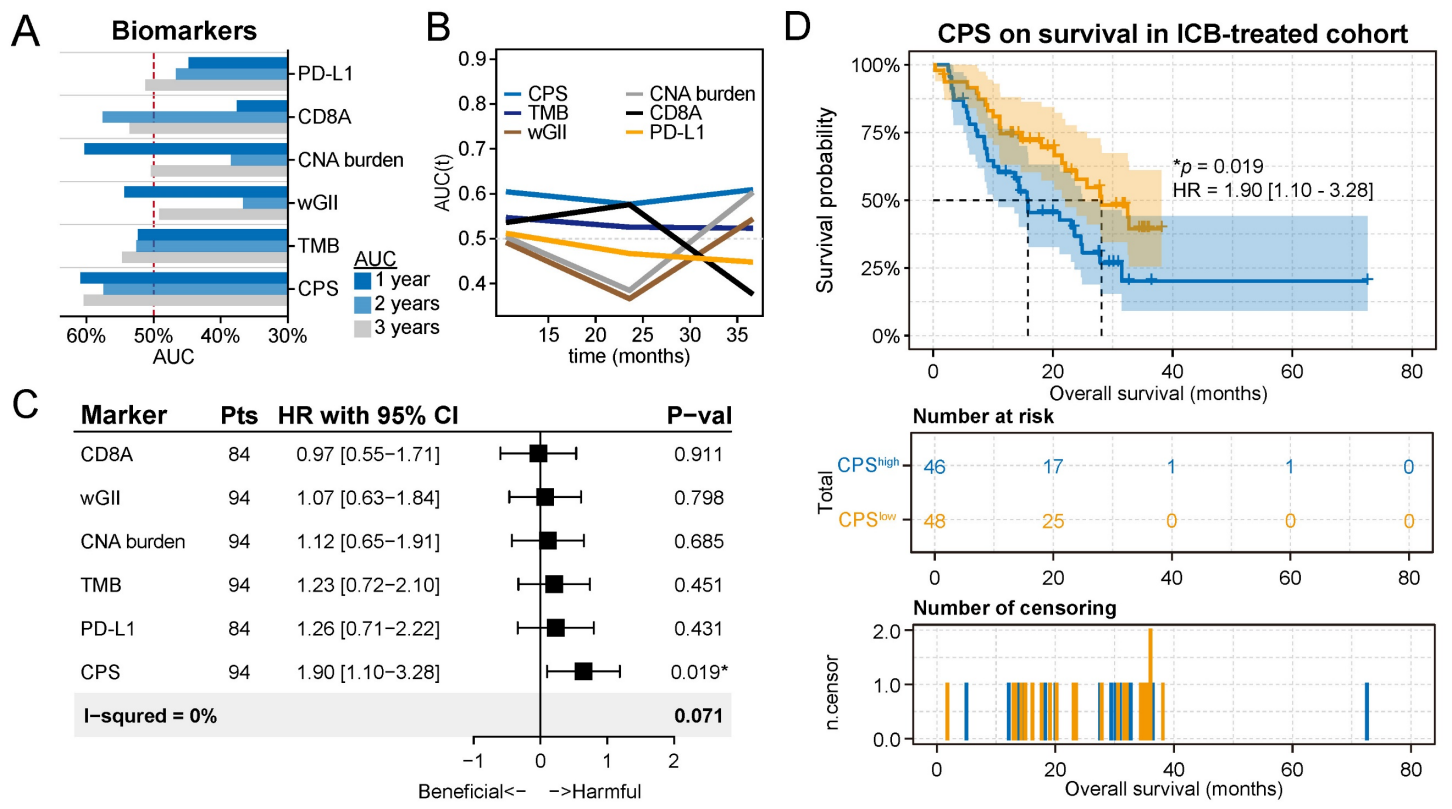
** Chromothripsis score predicts survival outcomes for patients after immunotherapy. (A)** Statistics of multiple biomarkers (including chromothripsis score (CPS), tumor mutation burden (TMB), weighted genome instability (wGII), copy number alteration (CNA) burden, expression of CD8A, and expression of PD-L1) based on 1-, 2-, and 3-year area under the curves (AUC)s. **(B)** Curves of AUCs over time for multiple predictors. **(C)** Results of univariate Cox regression analyses using the biomarkers for all solid cancers. Forest plots showing the log_2_ hazard ratio (95% confidence interval). ∗Adjusted p < 0.05. **(D)** Kaplan-Meier curves of overall survival in the integrated immunotherapy dataset. Patients were classified into CPS^high^ or CPS^low^ groups according to the median value of the CPS.

We compared the sequential trends associated with these predictors in terms of overall survival (OS) by generating time-dependent receiver operating characteristic (ROC) curves (**Figure 5A**). The time-dependent ROC curve for CPS was continuously superior (**Figure 5B**). According to the survROC curves for 1-, 2-, and 3-year OS for CPS, the ROC curve was found to be greater for 1- and 3-year OS (**Figure S4**). The univariate Cox regression analysis showed that CPS was significantly associated with survival in patients treated with ICB immunotherapy (HR with 95% CI = 1.90 [1.10-3.28], *P* = 1.9 × 10^-2^, **Figure 5D**). The remaining predictors did not show significance, which is consistent with previous results.

We then defined patients with CPSs greater than the median as the CPS^high^ group, and these patients had a median survival time of 15.8 months, which was significantly lower than the median survival time of 28.1 months for patients in the CPS^low^ group (*P* = 1.9 × 10^-2^, **Figure 5D**). Grouping patients again using the median as the threshold, it was found that other biomarkers could not be adequately used to classify patient responses in these cohorts (**Figure S5**). Furthermore, we observed a numerical trend towards lower response rates and objective response rates in the CPS^high^ group compared to the CPS^low^ group (*P*=0.64, Fisher exact test, **Figure S6A**; *P*=0.48, Fisher exact test, **Figure S6B**). These results support the potential of CPS to complement established biomarkers in identifying patients who are likely to respond favorably to immunotherapy.

## Discussion

The role of chromothripsis in anti-tumor immunity is unclear, even though it is prevalent in tumors and plays an important role in tumor evolution. Here, we report that chromothripsis is associated with reduced cytotoxic immune cell infiltration and that its copy number signature-based score can be used to reliably predict survival outcomes for patients receiving ICB treatment. In both discovery and validation datasets, we found that immune infiltration in tumors with chromothripsis was reduced and that immune suppression in the tumor microenvironment was enhanced. These results all suggested an unfavorable survival outcome for patients harboring tumors with chromothripsis receiving immunotherapy.

Chromothripsis is involved in the cGAS-STING signaling pathway through micronucleus formation. Activation of the cGAS-STING signaling pathway in innate immune cells induces the production of type I interferon, which initiates an antigen-specific immune response leading to tumor killing 45, 46. Recent findings suggest that STING can induce regulatory B cells to suppress the anticancer capacity of NK cells 47. Thus, the role of STING in immunotherapy is controversial 48.

The frequencies of mutations in ICB-responsiveness-related genes (including *Kras*, etc.) and the CNV burden were not consistent in both the discovery and validation datasets. One possible explanation for this is that chromothripsis is heterogeneous. Chromothripsis could exhibit certain chromosomal preferences; for example, chromothripsis is enriched in chromosome 12 in liposarcomas and chromosomes 3 and 5 in kidney renal cell carcinomas 7. Meanwhile, the location of chromothripsis occurrence might be different among different patients with the same type of cancer, which provides diverse options for cancer evolution.

TMB symbolizes the intrinsic characteristics of tumor and is representative of immunogenic neoantigens 40, 49. We found that chromothripsis was associated with a high TMB, which seems to create a contradiction. One possible explanation for this is that in our study, both antigen presentation and antigen recognition were impaired in tumors with chromothripsis, which resulted in the inability of tumor neoantigens to exert their conventional effect on anti-tumor immunity. In fact, CIN leads to impairments in antigen processing and presentation and has been described in the previous results 40, 50. In addition, it has been reported that TMB is not an accurate predictive biomarker for ICIs, for example, non-small cell lung cancer patients with KRAS and SKT11 co-mutations and high TMB do not respond to immunotherapy 51. This could also explain the association of patients with chromothripsis with high TMB but with poor responsiveness to immunotherapy.

We further integrated multi-omics and clinical data from multiple published clinical trials of ICB. Both time-dependent ROC curves and univariate Cox regression analysis showed that the CPS, based on a copy number signature, outperformed other biomarkers. Chromothripsis, as a potential indicator, may thus predict survival outcomes for patients after ICB immunotherapy.

In conclusion, our analysis suggests that the identification of chromothripsis could be useful to determine which patients are most likely to respond to ICB immunotherapy. Furthermore, an in-depth study of the mechanisms by which chromothripsis affects anti-tumor immunity and responses to immunotherapy might provide a pathway that could be therapeutically targeted to improve response rates to ICB. With the accumulation of available samples in the future, more comprehensive and in-depth studies will be possible.

## Materials and Methods

### Tumor microenvironment analysis

Bulk RNA-seq data originated from the TCGA and PCAWG databases. We acquired feature gene panels for 28 immune cell types from a publication 24. Meanwhile, the relative abundance (represented by enrichment scores) of the immune cell types in tumor microenvironment was quantified by Single Sample Gene Set Enrichment Analysis (ssGSEA) 52. Similarly, we used multiple gene signatures from clinical trials or widely used gene signatures to quantify the ICB-responsiveness of the tumor microenvironments by performing ssGSEA. The enrichment score of the ssGSEA had a negative value, which resulted in the relevant ratios of the relative abundance of the relative cells not being calculated. Considering this situation, we replaced the ssGSEA score with the geometric mean, calculated using the gene signatures of each cell type, which ensured that the calculation of the relative ratios was correct.

GSVA, as an unsupervised gene enrichment method, enables modeling in highly heterogeneous sample populations to estimate related variation in pathway activity. We quantified the activity of signaling pathways (including antigen processing and presentation, CD8 TCR downstream pathways, IL-2 signaling, IL-15 signaling, NK-mediated cytotoxicity, and IFN-γ pathway) that were derived from MSigDB gene sets and involved anti-tumor immune effects. GSEA, which is also a gene set enrichment method, was used to validate the GSVA results 52, 53.

### Genetic features of cancer

VCF or MAF files containing somatic mutations and copy number alteration profiles were downloaded from TCGA and PCAWG. Using the R package Maftools (version 2.8.05), we converted VCF files to MAF files for the subsequent statistical analysis 54. The total number of somatic gene coding errors, insertions or deletions, base substitutions per million bases is defined as TMB which was calculated using the tmb function in the R package Maftools, based on the acquired somatic mutations.

Patients were divided into two groups, namely the chromothripsis and non-chromothripsis groups. Then, we determined the frequency of mutations in *TP53*, *KRAS*, *PTEN*, *JAK1/2*, and *B2M* in different groups. The burden of copy number alterations indicates the total number of genes with copy number gains or losses. Bedtools (version 2.30.0) 55 was utilized to overlap copy number alteration profiles with protein-coding regions to obtain the number of genes exhibiting gains and losses per copy number segment, and the results were finally summarized.

Tumor ploidy is expressed as a weighted median integer copy number (the weight is the length of the copy number segment). With tumor ploidy, we obtained the percentage of genomic material gained and lost per chromosome. Ultimately, the average of this percentage for all autosomes is the wGII of a sample 39. These approaches were also applied to the immunotherapy cohorts.

### Exome analysis pipeline

Raw sequencing data originated from the immunotherapy cohorts. Reads aquired by paired-end sequencing were alignd to the human reference genome (HRCh38) to obtain BAM files (via Burrows-Wheeler Aligner (BWA, v0.7.17) 56). Further, based on the BAM files, we completed further indel realignment, base-quality score recalibration, and duplicate-read removal bythe Genome Analysis Toolkit (GATK, version 4.2.5.0) 57. We annotated the mutations by ANNOVAR (build 2020-06-08) 58.

Processed paired BAM files (tumor and matched normal samples) were used as input to MuTect2 (which is integrated in GATK, default parameters) to identify somatic single nucleotide variants and small insertions or deletions. We further filtered the acquired mutations based on three rules. First, we filtered for high-confidence variants (coverage of at least 5-fold or allele ratio > 0.05). Second, non-silent variants (including missense, nonsense, frameshift, and splice site variants) were selected. Third, only rare variants (the frequencies of variants must be less than 0.005 in relevant databases, including 1000G, ESP6500, dbSNP, ExAC) were selected. loss-of-heterozygosity events and CNAs were acquired by Fraction and Allele-specific Copy number Estimate from Tumor/normal Sequencing (FACETS) 59.

### CNV signature analysis

Based on the processing protocol in the publication 13, we further processed copy number profiles from the TCGA, PCAWG, and immunotherapy cohorts, with processing details including the removal of regions corresponding to IgK, IgL, IgH, and X chromosomes, and exclusion of CN changes less than 50 kB. Similarly, we followed the definition of six essential characteristics of CN in the publication: 1) the size of the segments, 2) the absolute CN of the segment, 3) the CN difference between adjacent segments, 4) the number of breakpoints per chromosome arm, 5) the length of oscillating CN segment chains, and 6) the number of breakpoints per 10 Mb. Based on the mclust R package, we identified the optimal number of categories for each CN feature. The hierarchical Dirichlet process (hdp) was utilized to perform de novo CN signature extraction and was performed based on the CN category matrix. The extracted CN signatures were used to calculate the prediction metrics of chromothripsis using the generalized linear model. Based on area-under-the-curve from ROC curves, we evaluated the prediction accuracy of chromothripsis via 10-fold cross-validation.

### Analysis of chromothripsis

For whole exome sequencing data, the obtained copy number variation profiles were used for CNV signature analysis. First, we filtered the CNV profiles and removed the corresponding regions on X chromosome. The optimal number of categories was obtained by clustering the six CN basic features in the CNV profiles. Then we extracted the CN features from scratch by the hierarchical Dirichlet process (hdp), and these features were input to the established CN signatures prediction model (generalized linear model) as basic elements to obtain the prediction metrics of chromothripsis, which are CPSs.

### Survival analysis

To ensure comparability among individual indicators, CPSs obtained from the CN feature prediction model were grouped in the same way as other indicators (including TMB, wGII, CNA burden, PD-L1 expression, and CD8A expression). The median of all indicators was used as a threshold to group patients, and the one higher than the median of this indicator was the high group. Univariate Cox regression analysis was applied to determine HRs. Kaplan-Meier analysis was employed to estimate survival and log-rank test was used to determine the p-values.

### Statistical Analysis

Our analysis was performed with R version 4.0.5. The Fisher's exact test was used for 2 × 2 tables of categorical variables and Wilcoxon rank-sum test was applied for differences in continuous variables unless otherwise specified. The noted software tools (including GSVA, pROC, Maftools, hdp, glmnet, timeROC, and survminer) that we used throughout the analysis are publicly available.

### Data and materials availability

All data are available in the main text or the supplementary materials. All raw data used in our analysis are publicly available. We acquaired the datasets for clinical parameters, SCNAs (SNP-array-based data), mutations, and RNA-seqfrom TCGA database (https://tcga-data.nci.nih.gov). The datasets for clinical parameters, SCNAs (the next-generation sequencing-based data), mutations, and RNA-seq came from the PCAWG database (https://dcc.icgc.org/pcawg/). To ensure the reliability of the hierarchical Dirichlet process, we integrated all immunotherapy cohorts to expand the sample size. For immunotherapy cohorts, we utilized raw whole exome sequencing data and RNA-seq clinical parameters from the following studies: 1) Hugo et al., an advanced melanoma anti-PD-1 treated cohort; 2) Riaz et al., an advanced melanoma anti-PD-1 treated cohort; 3) Zhao et al., an advanced glioblastoma anti-PD-1 treated cohort. RECIST 1.1-based quantifications of responses were then used to designate patients as responders (stable disease (SD) for ≥ 6 months, partial response (PR), or complete response (CR)) or non-responders (SD with <6-month duration or progressive disease (PD)). Similarly, patients could also be distinguished as objective (CR or PR) or non-objective (PD or SD) responders. In TCGA and PCAWG databases, the types of tumor samples include fresh tissue, liquid nitrogen cryopreserved tissue, dry ice cryopreserved tissue, or paraffin tumor tissue. In the immunotherapy cohort, Zhao cohort did not specify the type of tumor sample, and both Riaz and Hugo cohorts included fresh tissue samples.

## Supplementary Material

Supplementary figures and tables.

## Abbreviations

ICB: immune checkpoint blockade
CPSs: chromothripsis scores
CIN: chromosomal instability
SCNA: somatic copy number alteration
ecDNA: extrachromosomal DNA
CAN: copy number alteration
TCGA: the Cancer Genome Atlas
MDSCs: myeloid-derived suppressor cells
TAMs: tumor-associated macrophages
Tregs: regulatory T cells
CD8^+^ T cells: cytotoxic T cells
NK cells: natural killer cells
GSVA: gene set variation analysis
GSEA: gene set enrichment analysis
PCAWG: pancancer analysis of whole genomes
WGS: whole genome sequencing
SV: structure variantion
TMB: tumor mutation burden
wGII: weighted genome instability
WES: whole-exome sequencing
OS: overall survival
ROC: receiver operating characteristic
ssGSEA: single sample gene set enrichment analysis
