# Supplementary material for: Chromothripsis is correlated with reduced cytotoxic immune infiltration and diminished responsiveness to checkpoint blockade immunotherapy
Source: Theranostics. 2023 Feb 27;13(4):1443–53. doi: 10.7150/thno.81350 (PMC10008737; doi:10.7150/thno.81350)
Supplement: Supplementary file 1 — Supplementary figures and tables. [file thnov13p1443s1.zip › sup_figs.pdf]

Supplementary Materials for

Chromothripsis is correlated with reduced cytotoxic immune infiltration and diminished responsiveness to checkpoint blockade immunotherapy

Han Chu *et al.*

*Correspondence to Qingzhu Jia (E-mail: qingzhu.jia@tmmu.edu.cn), Bo Zhu (E-mail: bo.zhu@tmmu.edu.cn), and Haoyang Cai (E-mail: haoyang.cai@scu.edu.cn).

This PDF file includes:

Figures. S1 to S6

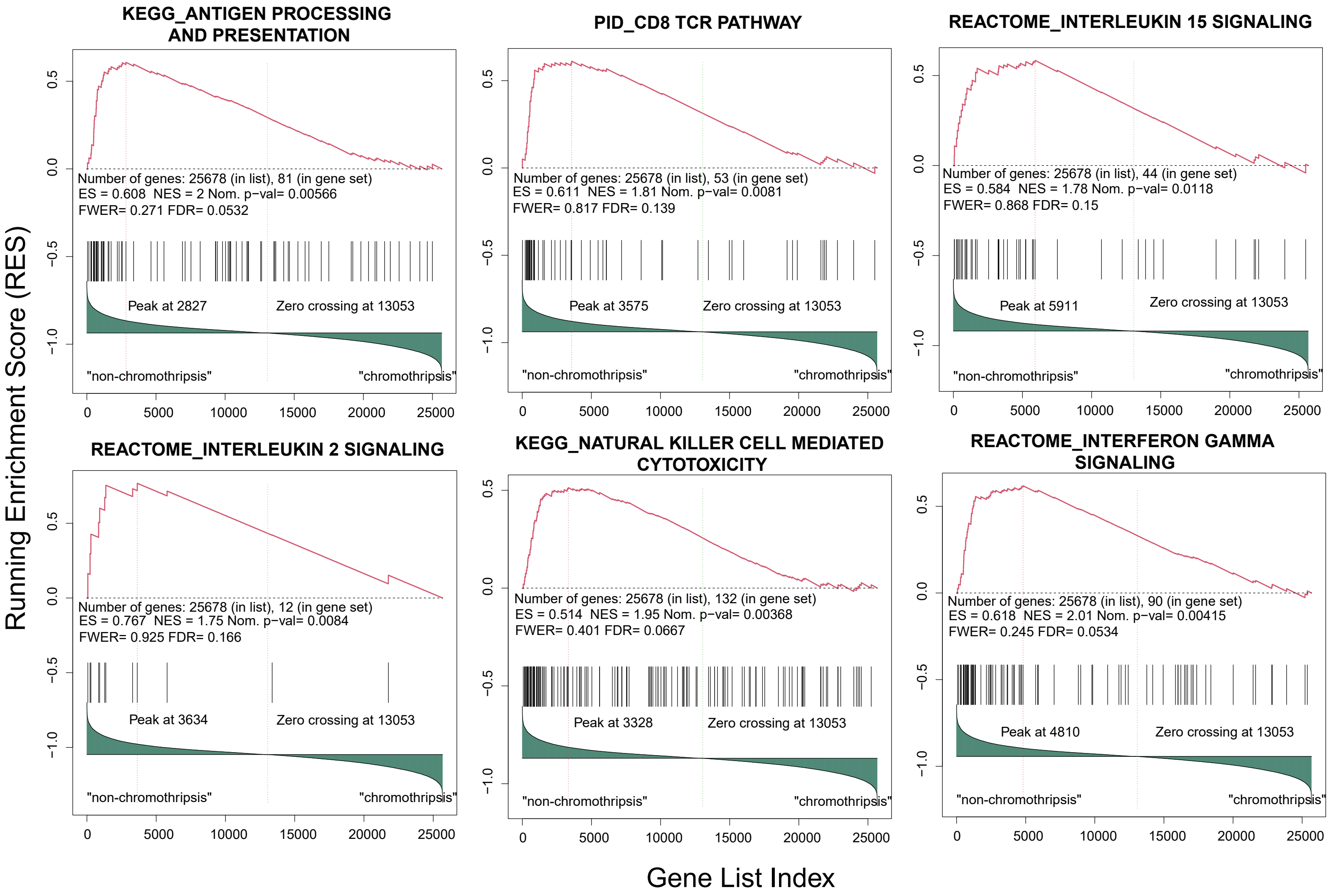

Figure S1. Gene set enrichment analysis (GSEA) showing the enrichment of pathways related to anti-tumor immunity in tumors without chromothripsis in TCGA dataset.

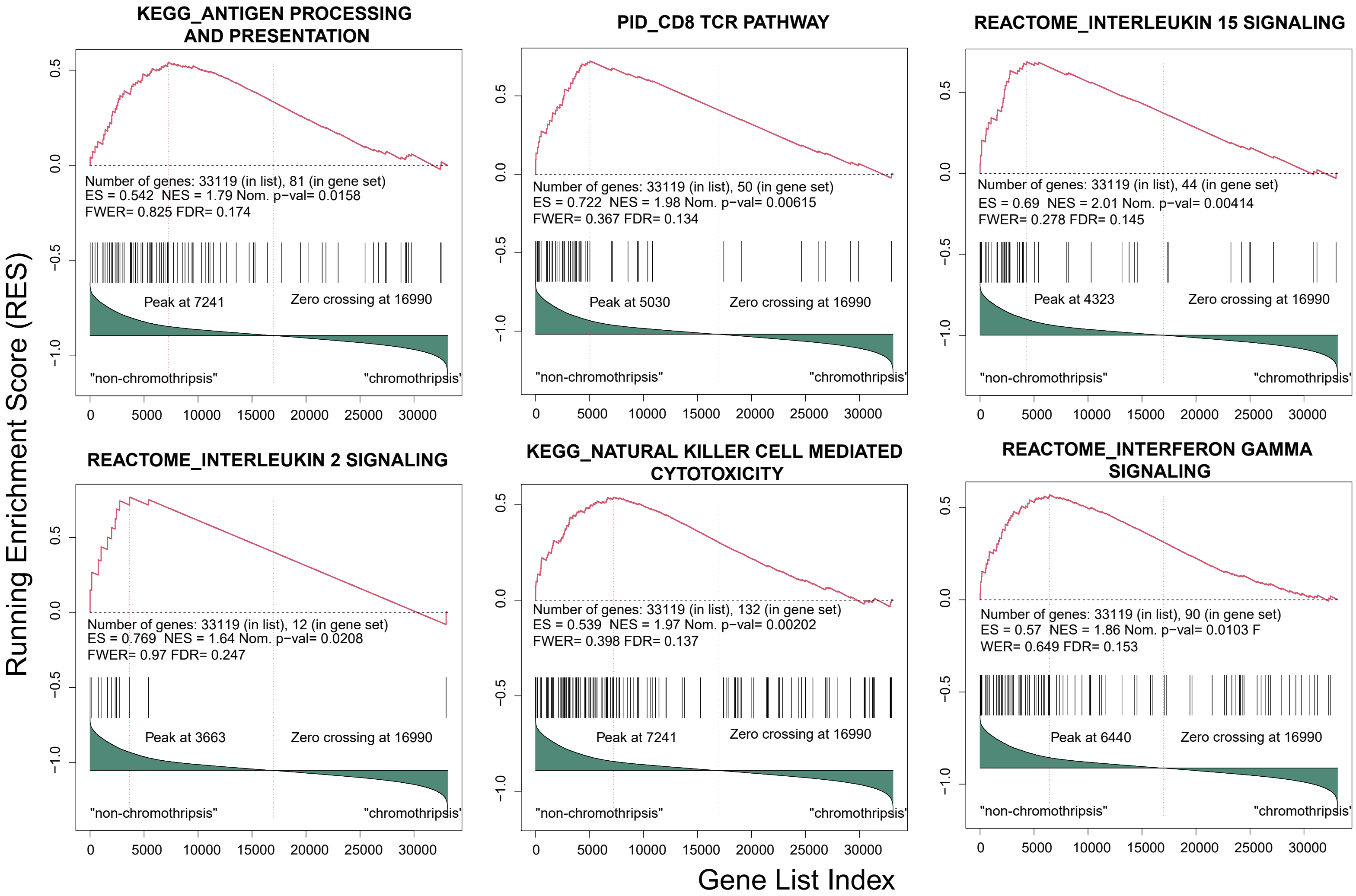

Figure S2. Gene set enrichment analysis (GSEA) showing the enrichment of pathways related to anti-tumor immunity in tumors without chromothripsis in the PCAWG dataset.

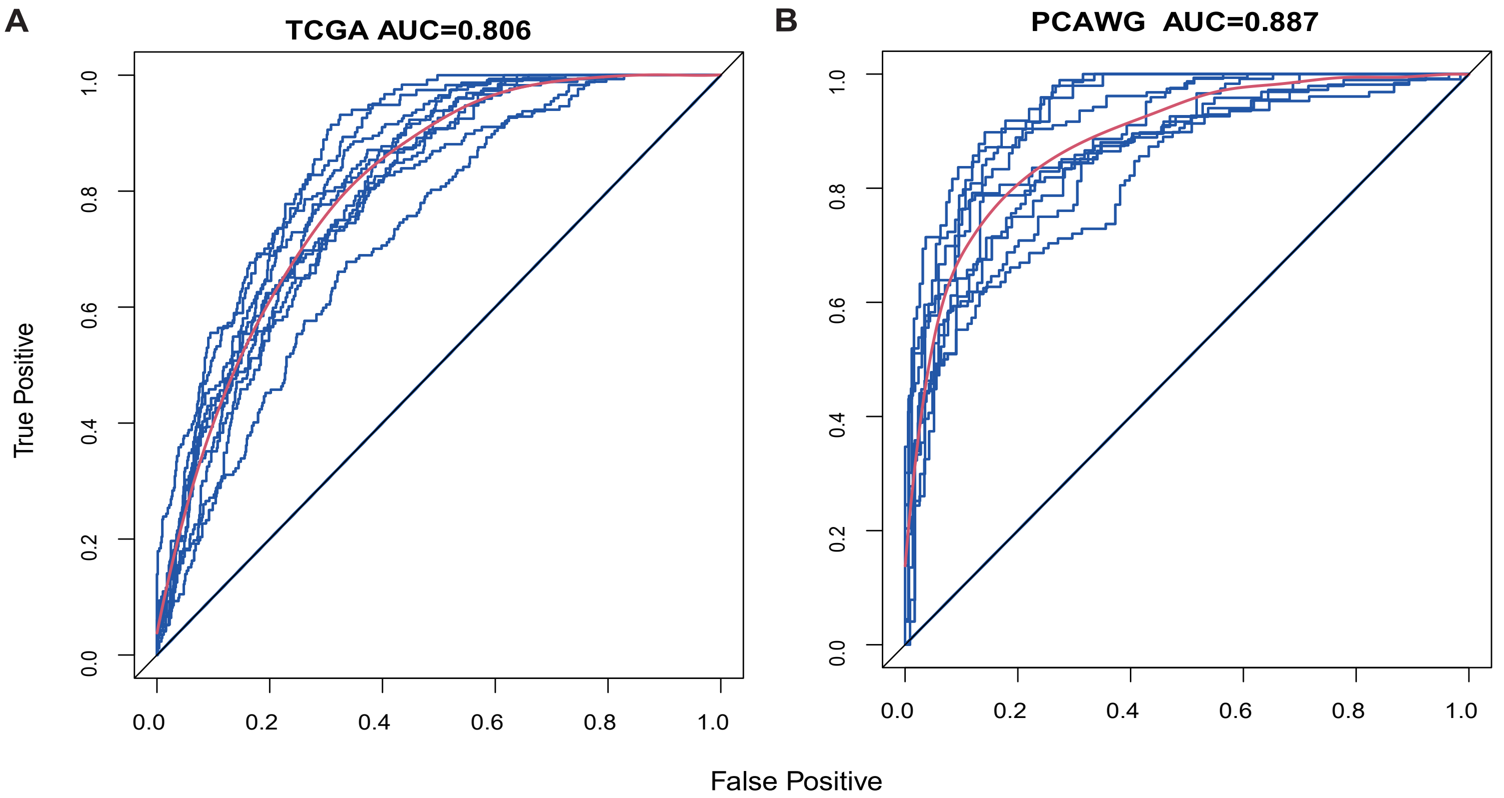

Figure S3. Receiver operating curve (ROC) for the prediction of chromothripsis based on the copy number signature prediction model from **(A)** TCGA dataset and **(B)** the PCAWG dataset. Blue lines represent individual ROC values from 10-fold cross-validation, red lines represent the means of individual ROC values, and AUC is the mean area under the curve.

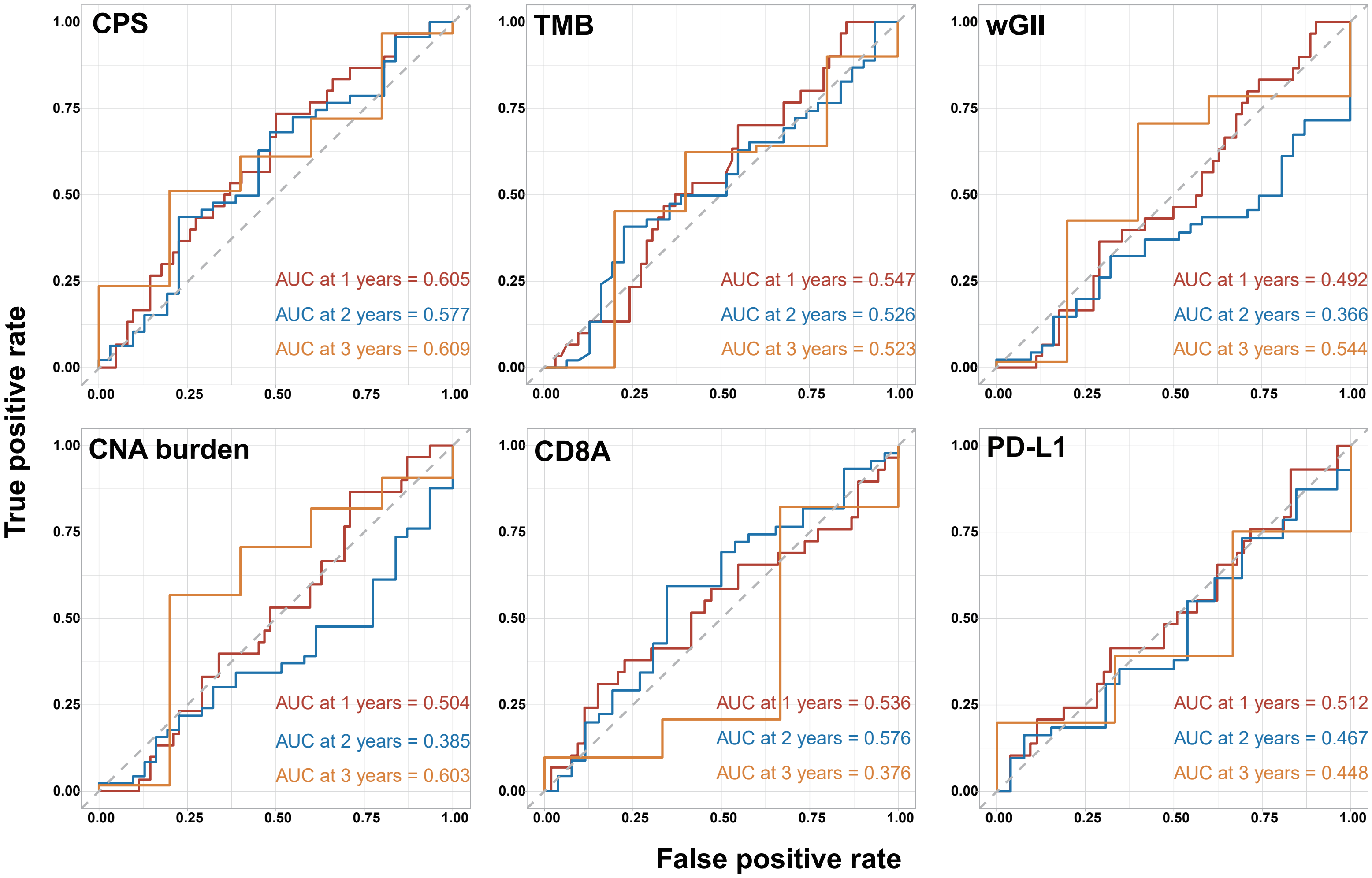

Figure S4. Time-dependent receiver operating characteristics for 1-, 2-, and 3-year OS for CPSs, tumor mutation burden (TMB), weighted genome instability (wGII), copy number alteration (CNA) burden, expression of CD8A, and expression of PD-L1. Area under the curve values (AUCs) with 95% bootstrap confidence intervals are provided.

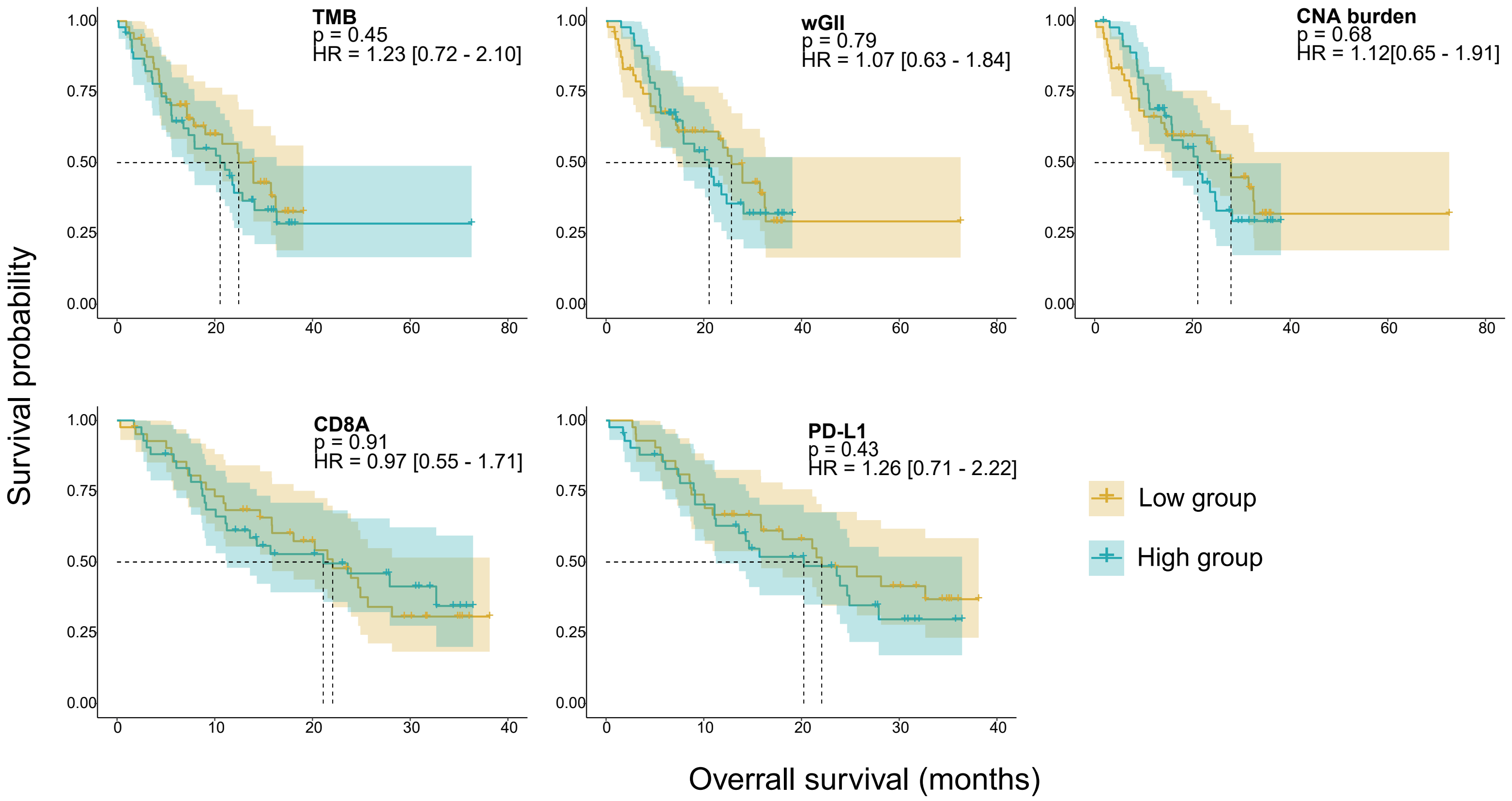

Figure S5. Kaplan–Meier curves of overall survival in the integrated immunotherapy dataset. Patients were classified into two groups according to the median value of biomarkers (including the tumor mutation burden (TMB), weighted genome instability (wGII), copy number alteration (CNA) burden, expression of CD8A, and expression of PD-L1).

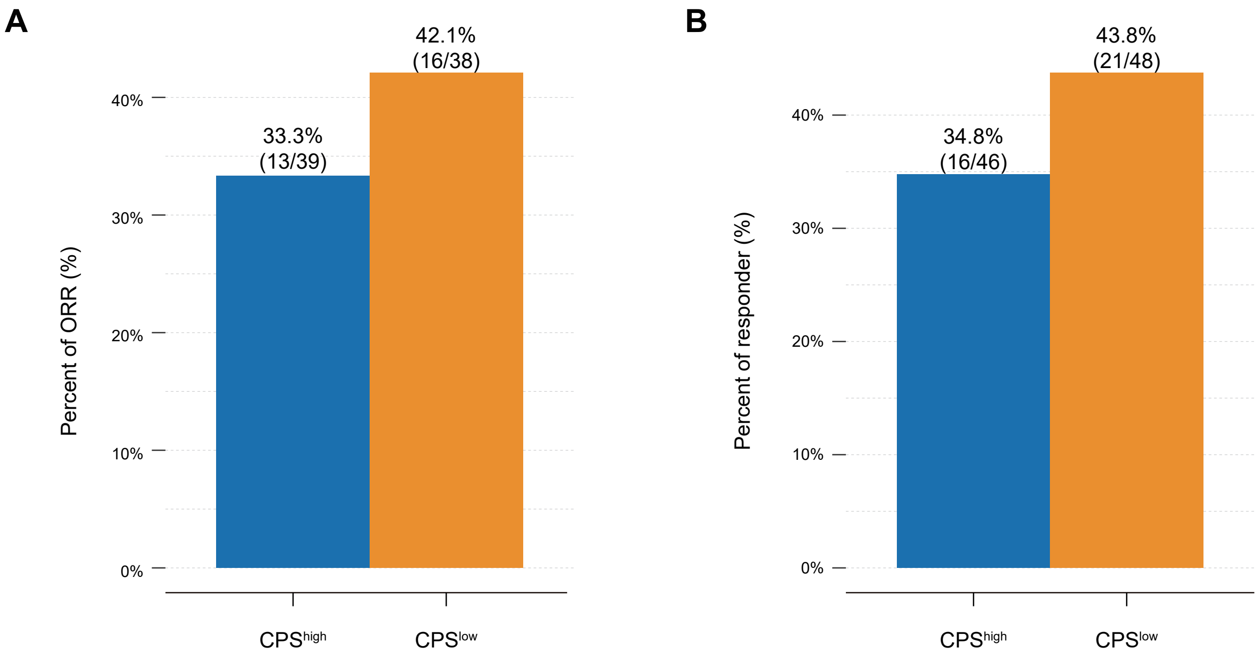

Figure S6. Objective response rates (A) and Response rates (B) in CPShigh or CPSlow groups in the integrated immunotherapy dataset.
